# Synergic effect of chronic hepatitis C infection and beta thalassemia major with marked hepatic iron overload on liver fibrosis: a retrospective cross-sectional study

**DOI:** 10.1186/1471-230X-4-17

**Published:** 2004-08-12

**Authors:** Farid Azmoudeh Ardalan, Mohammad RF Osquei, Mohsen N Toosi, Guiti Irvanloo

**Affiliations:** 1 Department of Pathology, Imam Khomeini Hospital, Tehran University of Medical Sciences, Tehran, Iran; 2 Department of Gastroenterology, Imam Khomeini Hospital, Tehran University of Medical Sciences, Tehran, Iran

## Abstract

**Background:**

Increased hepatic iron is assumed to potentiate progression towards liver fibrosis in chronic hepatitis C virus (HCV) infection. In this study we have evaluated the potentiating effect of marked hepatic iron overload and chronic HCV infection on hepatic fibrosis in thalassemic patients.

**Methods:**

Liver biopsies of one group of patients with beta thalassemia major and chronic HCV infection (group 1) was compared with two groups of patients (groups 2&3) with either chronic HCV infection or thalassemia major, respectively (20 patients in each group). Necroinflammation, fibrosis, and iron overload were graded and compared.

**Results:**

Stage of fibrosis in group 1 patients was significantly higher than the other two groups (p < 0.05). Necroinflammatory grade was significantly lower, but iron score was significantly higher in thalassemic patients (group 3) in comparison to groups 1 and 2 (p < 0.05).

**Conclusion:**

Our results indicate that marked liver iron overload and HCV infection in thalassemic patients have potentiating effect on hepatic fibrogenesis.

## Background

Thalassemia major is an inherited disorder particularly common in people of Mediterranean, African, and South-east Asian ancestry. It is characterized by decreased production of beta chain of hemoglobin. Clinical features result from anemia, markedly expanded marrow space, and transfusional and absorptive iron overload. In these patients iron overload is often inevitable, especially when iron chelating agents are not used properly [1]. Hepatic iron overload leads to different degrees of liver fibrosis, the severity of which is closely correlated with the severity of liver iron overload [2]. The pattern of iron deposition seen in the initial stages of thalassemia major is preferentially sinusoidal with a more or less diffuse distribution within the acinus. With significant loading, hepatocytes, bile duct epithelia, and fibrous tissue of portal tracts or septa will also show iron deposition [3].

HCV is responsible for 80–90% of post-transfusional cases of hepatitis in patients who have received blood transfusion(s) prior to the introduction of routine blood products screening in 1990 [4]. More than 75% of HCV infections become chronic and up to 20–30% progress to cirrhosis [3-5].

Increased hepatic iron may potentiate progression towards liver fibrosis in chronic HCV infection, and may contribute to poor response to interferon therapy [3,6,7]. Acceleration of hepatic fibrosis in patients with combined hereditary hemochromatosis (HH) and chronic hepatitis C infection has also been shown [7]. Nevertheless, the pattern of iron deposition in HH is initially hepatocellular and different from that of thalassemia major. In this study we have evaluated the potentiating effect of marked hepatic iron overload and chronic HCV infection on hepatic fibrosis in thalassemic patients. To the best of our knowledge this synergic effect has been studied just in another study on bone marrow transplanted thalassemic patients [8].

## Methods

A retrospective cross-sectional study was performed on sixty patients in three different groups as outlined below:

Group 1: Twenty patients (10 males, 10 females) with the diagnosis of thalassemia major and chronic HCV infection (BTM/CHI). The only risk factor of HCV infection in these patients was blood transfusion before 1990.

Group 2: Twenty patients (13 males, 7 females) with chronic HCV infection (CHI). The route of infection and the duration of disease were not known in most of the patients.

Group 3: Twenty patients (10 males, 10 females) with thalassemia major.

Thalassemia major was diagnosed by appropriate clinical and laboratory findings, and confirmed by hemoglobin electrophoresis. All the thalassemic patients had received multiple blood transfusions since childhood, and only those with at least moderate hepatic iron overload (2/4 based on Marx and Sindram hepatic iron scoring [9]) were included.

CHI was confirmed by positive anti-HCV (enzyme-linked immunosorbant assay), a positive HCV RNA by polymerase chain reaction and appropriate findings on liver biopsy.

Patients with history of alcohol intake, smoking, positive HIV serology, positive HBs antigen or HBc antibody, and other liver diseases (e.g., autoimmune hepatitis, drug hepatotoxicity, Wilson's disease, alpha-1 antitrypsin deficiency, hereditary hemochromatosis) were excluded from all the three groups.

All of the patients whose liver biopsies were submitted to the Central Pathology Department of Imam Khomeini Hospital (a Terhan University of Medical Sciences affiliated hospital) between April 2001 and January 2004 were retrieved. During this period, there were only 20 thalassemic patients with moderate to marked hepatic iron overload, so all of them were included in our study. Since the sex and age distribution of patients with thalassemia major and BTM/CHI were relatively the same, 20 patients with BTM/CHI and at least moderate hepatic iron overload were randomly selected from all the patients with this diagnosis. On the other hand, patients with CHI had a considerably higher age, so to match the age of the patients, we selected 20 of the youngest patients.

For all the sixty patients, we had hematoxylin and eosin, Masson's trichrome and Perls' Prussian blue stains. All the slides were reviewed by a single pathologist (FAA) who was blind to the diagnoses. The necroinflammation, fibrosis, and iron deposition were scored using modified Hepatitis Activity Index (HAI) grading, modified HAI staging, and Marx/Sindram hepatic iron scoring systems, respectively [9,10]. Presence or absence of macrovesicular steatosis was also assessed.

### Statistical analysis

The results are presented as mean value +/- standard deviation (for age), median and range values (for modified HAI grade, stage, and iron score) and percentages (for steatosis). Group comparisons were made using the two-tailed independent Student's t-test for age and two-sided Fisher's exact or Chi^2 ^tests for other variables with p < 0.05 considered to be significant.

For each patient with BTM/CHI (doubly exposed cases), there were two different unexposed cases, i.e. thalassemic cases (unexposed to HCV) and CHI cases (unexposed to iron overload). Considering "disease" as higher stages of fibrosis (modified HAI stage ≥ 3), our guesstimate of the expected frequency of disease in unexposed and exposed patients were about 25% and 75%, respectively. So the sample size was calculated as about 20 patients for each group with confidence level of 95% and power of 80%.

## Results and Discussion

The results of the study are summarized in table 1. This study supports the synergic effects of CHI and marked hepatic iron deposition in thalassemic patients on liver fibrosis. The patients with BTM/CHI had higher stages of liver fibrosis in comparison to patients with either CHI or thalassemia major alone. The patients with BTM/CHI had higher stages of fibrosis despite lower scores for iron overload in comparison to thalassemic patients. The potentiating effect of hepatic iron overload and CHI has been shown in other studies [7,8,11,12]. The cause of iron overload in the majority of studies has been hereditary hemochromatosis or *HFE *mutations.

**Table 1:** Demographic and histopathologic findings in the three groups

	**Group 1**	**Group 2**	**Group 3**	**P value**	
				
				**Group 1 vs. 2**	**Group 1 vs. 3**
Age(years)*	21.55+/-4.74	29.7+/-9.16	18.7+/-4.45	***0.001***	0.057
**Sex (M/F)**	10/10	13/7	10/10	0.33	1
**Modified Stage**^‡^	3 (1–6)	2 (0–6)	2 (1–4)	***0.02***	***0.01***
**Modified HAI grade**^‡^	5 (1–8)	6 (2–11)	2 (0–6)	0.36	***0.001***
Periportal inflammation^‡^	1.5 (0–3)	2 (0–4)	0 (0–2)	0.77	***0.004***
Confluent Necrosis^‡^	0 (0–1)	0 (0–2)	0 (0–1)	0.13	0.091
Focal Necrosis^‡^	1 (0–2)	2 (1–2)	1 (0–2)	0.10	0.45
Portal Inflammation^‡^	2 (0–3)	2 (1–4)	1 (0–2)	0.87	***0.002***
**Iron Score**^‡^	3 (2–4)	0 (0–1)	4 (2–4)	***0.000***	***0.024***
**Steatosis (%)**	10	20	0	0.66	0.48

* mean+/-standard deviation ^‡^Median (minimum-maximum) Group 1: Patients with beta thalassemia major and chronic HCV infection Group 2: Patients with chronic HCV infection Group 3: Patients with beta thalassemia major

Angelucci et al showed for the first time the role of iron overload and HCV positivity as independent risk factors for hepatic fibrosis progression in thalassemic patients following successful bone marrow transplantation [8]. The Angelucci's study has a few advantages over our study: firstly, serial liver biopsies have been studied and the rate of liver fibrosis progression assessed. Secondly, hepatic iron concentration was used instead of scoring of stainable iron. Thirdly, two pathologists reviewed the slides independently. And finally, the sample size was much larger, but that study does not show whether the rate of hepatic fibrosis progression in BTM/CHI patients is greater than patients with only CHI.

The potentiating effect of iron overload and HCV infection can be explained by the fact that both these agents produce oxidative stress in the liver [7,13]. In our study the grade of necroinflammation in BTM/CHI patients was not significantly different from CHI patients. Other studies have shown the same results [3,7].

Limitations of our study were that it was a retrospective study with limited number of patients in each group. The duration of HCV infection and genotype of virus were not known in our patients. The routes of infection in most of the CHI patients were not known and where probably different from our thalassemic patients. The age of our CHI group was significantly higher than the other two groups. Since older age at the time of infection is considered to be a risk factor for progression of chronic hepatitis C, the stage of fibrosis should have been higher in our CHI patients than BTM/CHI patients if iron had not had any potentiating effect on liver fibrosis. Our study was performed on thalassemic patients with moderate to severe liver iron deposition. At these stages of iron overload, the pattern of hepatic iron deposition in HH and thalassemic patients are nearly the same. So this study does not reveal the effect of only sinusoidal iron deposition on progression of fibrosis in HCV infected thalassemic patients.

## Conclusions

Our results show that moderate to severe liver iron overload and chronic HCV infection in thalassemic patients have potentiating effect on hepatic fibrogenesis. So the proper use of chelating agents in HCV-infected thalassemic patients seems to be of great importance in delaying progression of the liver disease. The presence of liver siderosis has been shown to be related to poor response to interferon alpha (IFN) in non-thalassemic patients [3,6,7,12,14,15]; hence one can expect a poor response to IFN therapy because of transfusion related siderosis in thalassemic patients. However studies have shown that in thalassemic subjects, there is a promising response to IFN therapy (as high as 50% sustained response in some series) [16,17]. Perhaps in these patients (BTM/CHI), the therapeutic protocol for chronic HCV infection should differ from those without significant iron overload, but further studies are needed to confirm or refute these suggestions.

## Competing interests

None declared.

## Authors' contributions

FAA reviewed all of the slides, participated in study design and statistical analysis of results, and prepared the manuscript. MRFO assisted in collection and entering of the data, cooperated in reviewing slides, statistical analysis of results, and preparing the manuscript. MN was the clinical consultant. GI reviewed some of the problematic slides. All authors read and approved the final manuscript.

## Pre-publication history

The pre-publication history for this paper can be accessed here:


